# Report of Four Cases of Compound Fracture, from the Pennsylvania Hospital

**Published:** 1840-03-28

**Authors:** 

**Affiliations:** Attending Surgeon


### CLINICAL REPORTS.

*Report of four cases of Compound Fracture, from the Pennsylvania Hospital.* Dr. RANDOLPH, Attending Surgeon.

[Reported by J. F. MEIGS, M. D. Resident Surgeon.]

*Case of Compound Fracture of the Thigh in a boy aged 15 years—cure at the end of five months.*

W. P., a coloured boy, aged 15 years, of spare frame, usually strong and healthy, was brought into the hospital on the 6th May, 1839. A very little time before, he was violently crushed between a large heavy barrel, and a dray, to which was attached a horse at full speed.

He was at first much prostrated, but soon reacted under the use of sinapisms, and a small quantity of wine. The femur was fractured a few inches above the condyles, and the upper fragment protruded through a ragged, irregularly circular wound on the outside of the thigh, about three inches above the extremity of the condyle. Another wound involving only the integument, and about three inches long, was situated upon the middle inner surface of the thigh. Considerable hæmorrhage took place from the internal saphena vein, which it was thought proper to secure by a ligature; no other bleeding of any consequence. The ends of the bone being placed as nearly in apposition as possible, both wounds were slightly drawn together by adhesive strips, lint, and a roller applied, and the limb placed in Physick's modification of Desault's splints. Ordered Tr. Opii. gtts. xxx.

The patient was visited by Dr. Randolph, who determined, in view of the age, and apparently good constitution of the boy, to attempt to save the limb.

*May 7th.*—Has passed a restless night, complaining much of pain. Pulse one hundred, weak; skin hot and dry; tongue moist, with a light coat of whitish fur; no stool; not having passed any urine, the catheter was resorted to. Dressings untouched.

℞.—Pulv. Doveri grs. vii., morning and evening. ℞.—Mist. Efferves. ℥vi. Ant. Tart. gr. i. Ft. sol. S. c. m. q. b. h. Mucilaginous diet.

*8th.*—Rested well; passed his urine voluntarily; one stool. Pulse one hundred, remains weak; skin hot and dry; tongue moist, with a thicker coat of fur; strength moderate. Diet and remedies as before. The roller was removed, having become too light from the swelling of the limb; other dressings not disturbed. A bandage of Scultetus in place of the roller, from the foot upwards.

In the evening, there being some prostration, a small quantity of wine whey was administered. Mist. Efferves. to be suspended. Pulv. Doveri continued.

*9th.*—Has passed a good night, but little pain. Pulse one hundred and twenty-eight, stronger than before; skin and tongue remain as before. Dressings untouched.

*10th.*—Doing well; general symptoms as at last note; no sloughing; suppuration not yet established; dressed with flax-seed poultices, and the same splints as before.

*11th.*—Rests well at night; no pain. Pulse ninety-eight, much stronger; surface of natural temperature, soft; tongue moist, and but slightly furred; one stool daily; passes urine freely. Appetite improving. Bread, tea, and gruel for diet. Wounds discharging a healthy yellow pus, in moderate quantity. No sloughing to any extent. Dressed as before.

*14th.*—Doing well since last not. Pulse ninety-six, a little weak; skin warm and soft; tongue moist and furred; bowels open daily; no pain in thigh; appetite good. Diet, mutton soup, bread, milk, &c. Limb dressed twice a-day. Suppuration free; leg a quarter of an inch shorter than the opposite one.

17th.—General symptoms as before; wound on the inside of the thigh granulating well. That on the outside is extending upwards, thus increasing the surface of the bone laid bare; large discharge of pus from this. Now dressed with the simple cerate, compresses of dry lint to absorb the discharge, and a Scultetus bandage with the splints.

*23d.*—Pulse one hundred and four, becoming weaker; skin warm; tongue nearly clean, moist and soft; bowels quite regular; appetite good; rests well at night. From the extension of the wound on the outside of the thigh, the bone is laid bare in the space of about three quarters of an inch, retaining, however, its brilliant white colour. Suppuration very abundant, but healthy in character. No union, as yet, in the bone. Dressings continued. Diet, milk, bread, mutton-chops and soup.

*June 1st.*—The ulcers remain in the same state as when last described. The necessity of moving the limb frequently, in order to change the dressings, has been found to give great pain and discomfort to the patient, and has contributed materially, no doubt, to keep up the irritation and consequent discharge. To remedy this inconvenience, a wooden splint was procured, of length sufficient to extend from the ankle to the groin, and curved to fit the anterior surface of the limb. This being well padded with carded cotton, was secured above and below the seat of injury, by means of rollers. The dressings being applied, a third bandage was passed lightly over them, to keep them in place, the limb slightly elevated from the bed, and a towel or oil cloth filled with bran, placed beneath, to receive and absorb the discharge. By removing the last roller, the ulcers were readily cleansed without disturbing, in the least, the general line of the limb, thus preventing the laceration of the newly-formed granulations, which could not but take place in the old mode of dressing. The first and second rollers were changed but once a week, and when removed one at a time, did not cause much disturbance to the fracture.

From this time, there was a gradual and steady improvement, the health became good, the discharge constantly and regularly subsided; the ulcers granulated freely, and the exposed bone, which, notwithstanding its being constantly bathed in pus, had retained its pearly whiteness of appearance, was completely covered without any necrosis. The limb became firm, and when the splint was removed in the early part of July, the fracture had become almost firm, and there was only a small sinus, admitting a probe, and discharging a very little pus daily, remaining.

In August, he was attacked with erysipelas, followed by sloughing, and remained for a time in a very precarious state. Gradually, however, he recovered from this, and as the fracture remained perfectly firm, and the wounds had completely closed, he was allowed to get up, and walk about with the assistance of crutches, in the first week of October. On the 18th October, he was able to walk with one crutch and a cane.

The limb was shortened exactly two inches, but from the descent of the pelvis upon that side, he is able to walk without a high-heeled shoe. The mass of callus is very large, measuring three inches and a half in diameter, at the distance of two inches above the patella.

The general health of the patient became completely established, and he left the house on the 9th of November, 1839.

*Case of Compound Fracture of the Thigh in a child of* 11 *years—Fracture firm, with only a small ulcer remaining at the end of six months.*

J. C., aged eleven years, of healthy constitution, was brought into the hospital on the 11th of November, 1839, having fallen, a short time previously, the distance of about thirty feet, from a tree to the ground.

The femur was fractured in the middle of the diaphysis, and the upper fragment protruded through the outer edge of the rectus muscle, producing a ragged wound of about an inch in size; a piece of bone, an inch and a quarter long, composing a third of the circumference of the shaft, was found loose upon the clothes of the patient. Hæmorrhage very slight. No difficulty was found in reducing the fracture, and bringing the limb to nearly its proper length. Adhesive plaster, lint, and a bandage being applied over the wound, the limb was placed in Desault's splint, and very slight extension used.

*12th.*—Pulse one hundred and twenty; tongue moist and furred; skin hot and dry; no headach; passes urine freely; slept well. Diet, gruel and arrow root. Prescribed in the evening, Tr. Opii. gtts. xv. ℞.—Mist. Neutr. ℥vi., Ant. Tart. gr. ss. Ft. Solut. S. c. m. q. b. h.

*13th.*—Has not slept well; pulse as before; skin remains hot and dry; tongue furred but moist; no headach; no pain in the limb. Upon removing the dressings, the edges of the wound were considerably inflamed and tender to the touch. Ordered four ounces of blood to be taken from around it by leeches and a warm poultice to be applied. Diet as before. Tr. Opii. gtts. v. q. h.

*14th.*—Pulse one hundred and twenty-four, of moderate strength; tongue moist, fur disappearing; no pain; bowels open; has passed a very good night. Thigh much swelled in its whole extent. Wound looks well; a moderate discharge of healthy pus. Dressed with flax-seed poultice twice a-day, and the same splints as before.

*15th.*—Has passed a very good night. Pulse one hundred and twelve, of sufficient force; skin slightly warm, moist, and soft; tongue as before; no headach; one stool; no pain in limb.

*19th.*—Pulse one hundred and twenty, of moderate strength; skin warm and soft; tongue moist and but little furred. Prescribed yesterday half an ounce of castor oil, which operated freely. The tension of the limb from effusion is rapidly diminishing; very little pain. Dressings continued; diet, chicken-broth, gruel, and arrow root. Suspend mixture.

*24th.*—Continues to improve; pulse one hundred, of natural strength; skin of proper temperature, soft; tongue nearly natural; no headach; bowels slightly costive, for which oil is given from time to time; suffers but little pain; appetite good. Suppuration is now completely established; discharge of healthy character. Dressed as before.

*Dec. 11th.*—Doing well; pulse regular; skin soft, of proper temperature; tongue natural; rests well at night; bowels regular; appetite good; diet, chicken, oysters, coffee, bread, tea, &c. The swelling of the limb has nearly disappeared; the wound is now about an inch in diameter, circular, the edges somewhat indurated. From its centre projects the inferior extremity of the upper fragment, so that a quarter of an inch of its length is exposed, and is of a dark colour. Discharge amounts to about half an ounce in the course of the day. The leg is shortened three quarters of an inch. Dressed as before.

*Dec. 29th.*—General health very good. To-day finding that the exposed portion of bone was loose, it was removed by the forceps without any difficulty. The piece is about an inch and a quarter long, forming two thirds of the whole circumference of the shaft. The limb is shortened an inch and a half, with a very slight deviation from its proper line, from the ends of the fracture having passed each other slightly in front and behind. Union not yet firm, but becoming so. Still slight discharge of pus.

*March* 1*st*, 1840.—Since the date of last note, the fracture has gradually and firmly united; the ulcer is slowly contracting; the discharge has regularly decreased in quantity, until at present it amounts to a very few drops in the twenty-four hours. General health perfect; all his functions being regularly and properly performed. Two days ago the leg was removed from Desault's splints, and placed in a curved splint, fitting the bottom of the limb. Full diet, chicken, butter, oysters, bread, tea, &c.

*March* 30*th*.—Remains as before, except that the ulcer is of smaller size; no connection with the cavity of the fracture can be found, and from the nature of the discharge, it is not probable that there is any. Health very good. Dressed as before.

*Case of severe Compound Fracture of the Right Humerus, in a boy of* 15 *years—complete cure in five months.*

R. T., a boy aged 15 years, enjoying good health, though of rather slight frame, was brought into the hospital on the 23d October, 1839. He was playing a few hours previously with a metallic flask, containing about half a pound of powder, when, in pouring a portion on the fire, the whole exploded. The flask was broken into several pieces, one of which struck him on the right arm a little above the insertion of the deltoid muscle, severing the skin, the belly of the biceps, a portion of the outer head of the triceps, and the humerus, laying bare the upper fragment of the bone for the space of about an inch. The median nerve was exposed, but neither the bronchial artery nor vein were injured. The right hand, which held the flask at the time of the accident, was unhurt, except by a slight contusion of the thumb. The hæmorrhage was considerable, but no artery could be found, and the blood ceased to flow, except by a rather abundant oozing for some hours after. The arm was placed in a curved splint, and the edges of the wound made to approach within an inch of one another by means of adhesive strips.

24*th*.—Prescribed last evening, Mist. Neut. ℥vi., Morph. Sulph. gr. i. Ft. sol. c. m. q. p. b. Patient rested well; had vomited several times yesterday, after taking laudanum; none after the above mixture; no pain in arm when at rest; cloths show slight oozing of blood. Pulse ninety, of good volume; skin of body warm; head warmer; no headach.

27*th*.—Doing well; rests well; pulse one hundred, of good strength; skin of moderate warmth; expression natural; free suppuration of yellow pus, with pultaceous, fetid discharge; arm dressed by laying it in a curved splint of an obtuse angle at the elbow, extending from the shoulder to the ends of the fingers; the wounds slightly drawn together by adhesive strips; over this dry lint and and a Scultetus bandage.

*Nov.* 5*th*.—Continues to improve; no fever; sleeps well; suffers no pain; appetite good. The ulcer is now about four inches in breadth, and two and a half in the length of the arm; granulating freely; the upper fragment laps over the lower, and is exposed to the extent of half an inch; is of a dark, brownish colour, evidently necrosing.

24*th*.—Doing well; pulse regular and soft; skin natural temperature; tongue clean and moist; no headach; appetite good; bowels regular; sleeps well; taking usually Tr. Opii gtt. x. every night. The ulcer is contracting, granulations firm and healthy; the discharge lessening in quantity; no union of the bone as yet. Diet, chicken, bread, soup, butter, tea, &c.

*Dec.* 11*th*.—The exposed portion of bone was found to be loose this morning, and readily removed by a pair of forceps. It is an inch in length, and forms a section of the shaft of the humerus. The limb is now one inch shorter than the opposite, and of very nearly its natural shape. The ulcer has much diminished in size, being now two inches in breadth, and one and a quarter in the length of the arm. The motion at the shoulder is free, at the elbow, slight stiffening.

*Dec.* 26*th*.—Ulcer daily contracting in size; a small portion of dead bone, to be felt by the probe, upon the upper extremity of the inferior fragment. Union perfectly firm. Dressed with Turner's cerate; a bandage applied, and the patient allowed to walk about. Continue full diet.

*March* 8*th*.—Since last note, the patient has been walking about without any interruption to his good health. Within a few days a hard scab has dropped from off the seat of the ulcer, leaving beneath, a firm healthy cicatrix. The limb has regained all its usual motions, with the exception of a very slight stiffening at the elbow, from its being so long kept in the angular position. This however is rapidly disappearing, and he will no doubt regain perfect use of the arm.

He left the house entirely well on the 11th March, 1840.

*Case of Compound Fracture of the Forearm—ends of bones protruding—excision of a portion of each—complete cure in four months.*

G. D. a boy aged 12 years, of healthy and robust appearance, fell, on the 21st October, 1839, five or six feet from an apple tree, upon the ground. He threw his hands forwards, and received the whole weight of the body upon them. When brought into the Hospital a few hours after the accident, the left wrist upon examination, was found to be considerably swollen on its back, while a depression existed upon its anterior face. The radius was raised from its articulation with the carpus, about the third of an inch. By a few minutes steady extension from the hand, it was readily restored to its proper place. On the fore part of the right forearm, about three inches above the wrist, there were two lacerated wounds, separated from each other by a narrow strip of skin and muscle, half an inch wide; from these wounds protruded the upper fragments of the radius and ulna, about half an inch. All attempts to reduce them by extension, proving fruitless, Dr. Norris excised a small portion of each, by means of a pair of bone nippers, when they were replaced without any difficulty. The ulnar and radial arteries pulsated at the wrist. The arm was dressed with adhesive strips and dry lint, and then extended upon a straight splint; no hæmorrhage of any consequence. Low diet was ordered, with an opiate in the evening.

On the second day after his entrance, the arm was laid in a curved splint, and dressed twice daily, with a flaxseed poultice. On the third day there was a slight erysipelatous inflammation about the wounds, which disappeared in a few hours.

*November 4th.*—Whole forearm somewhat swollen; hand œdematous; considerable discharge of healthy pus from the wounds. There is a small point over the end of the ulna on the back of the arm, where the entegument being very thin, it is probable that the bone will protrude; to be poulticed and retained in curved splint. General symptoms very good. No fever; appetite natural. Diet, soup, bread, rice and tea.

11*th.*—Ulcers on front of arm fast diminishing in size; granulations firm and healthy; discharge small in quantity. Dressed with simple cerate, and straight splint.

18*th.*—Two days since the integument over the ulna on the posterior face of the arm, ulcerated, and through the opening were discharged several spicula of necrosed bone. Dressing continued. General health completely established. Has full diet, and is allowed to walk about.

From this time up to the date of his discharge, the arm was dressed as usual, the fracture firmly united. Small portions of dead bone were discharged from time to time, and the ulcers did not completely close, until the latter part of January. When he left the house on the 18th of February, 1840, the limb was somewhat shortened; a large mass of callus had been thrown out, rendering the arm perfectly firm; the motions of the wrist remained free, and in all respects his arm was likely to be as serviceable to him as before the injury.

